# Comparative effectiveness of Tai Chi, Baduanjin, and health education for knee osteoarthritis: protocol of a three-arm randomized controlled trial

**DOI:** 10.3389/fspor.2026.1750333

**Published:** 2026-05-18

**Authors:** Tao Zeng, Zi-liang Chen, Yao-hui Zhou, Wei-qi Liu

**Affiliations:** 1 Zhongshan Hospital of Traditional Chinese Medicine Affiliated to Guangzhou University of Traditional Chinese Medicine, Zhongshan, Guangdong, China; 2 Zhongshan Hospital of Traditional Chinese Medicine, Zhongshan, Guangdong, China

**Keywords:** Baduanjin, health education, knee osteoarthritis, randomized controlled trial, Tai Chi

## Abstract

**Background:**

Knee osteoarthritis (OA) is a leading cause of chronic pain and disability. With projections indicating 642 million affected individuals globally by 2050, identifying safe, scalable, and potentially disease-modifying non-pharmacological interventions is an urgent public health priority. This study aims to compare the clinical effectiveness of Tai Chi, Baduanjin, and a health-education programme in preserving joint function and quality of life for patients with knee OA.

**Methods/design:**

A single-centre, 48-week, comparative-effectiveness randomised controlled trial comparing Tai Chi, Baduanjin and health-education programme will be conducted at Zhongshan Hospital of Traditional Chinese Medicine. Eligible participants consists of adults ≥60 years of age with symptomatic and radiographic knee OA. Participants will be randomly assigned (1:1:1) using a computer-generated randomization sequence to one of three groups: (1) Tai Chi training, 5 sessions per week (60 min each) for 12 weeks; (2) Baduanjin training, 5 sessions per week (60 min each) for 12 weeks; or (3) health-education, 1 session per week (60 min each) for 12 weeks. Outcome assessors will be blinded to group allocation. The primary outcomes are the pain, stiffness and physical function subscales of the Western Ontario and McMaster Universities Osteoarthritis Index (WOMAC). Secondary outcomes include the Berg Balance Scale, 6 min walk test and SF-36 quality-of-life assessment. All measures will be assessed at weeks 12, 24 and 48.

**Discussion:**

By comparing two traditional mind-body exercises against an active control group over 48 weeks, this trial is designed to determine whether these interventions offer durable clinical benefits as standalone therapies or provide additive value. The findings are expected to provide high-quality evidence regarding scalable, low-cost non-pharmacologic strategies for managing knee OA, with potential implications for public health policy.

**Clinical Trial Registration:**

International Traditional Medicine Clinical Trial Registry (ITMCTR), http://itmctr.ccebtcm.org.cn/, identifier ITMCTR2025000409.

## Background

Knee osteoarthritis (KOA) is now the leading cause of global musculoskeletal disability, characterised by chronic pain, progressive functional decline and impaired quality of life (1). The Global Burden of Disease Study 2021 estimated that 595 million people worldwide had symptomatic osteoarthritis in 2020, and projections indicate a 74.9% increase in cases by 2050 compared with 2020, driven predominantly by population ageing and rising obesity (2). In economic terms, recent Nordic-wide registry data from the BISCUITS study show that the total annual incremental societal cost attributable to osteoarthritis—comprising direct medical expenditures and indirect productivity losses—averaged €3,224–4,969 (≈US$ 3,600–5,500) per patient in 2017 (3). Hence, safe, scalable and cost-effective non-pharmacological interventions are now an international priority (4).

Current pharmacological options for knee osteoarthritis exert only modest and transient symptom relief; non-steroidal anti-inflammatory drugs and acetaminophen frequently provide insufficient analgesia, while their chronic use is associated with appreciable gastrointestinal, renal and cardiovascular harms (5–7). Tai Chi and Baduanjin are increasingly recommended mind-body exercises for knee osteoarthritis. Although accumulating trials report symptomatic and functional benefits, heterogeneity in training parameters precludes definitive conclusions regarding their comparative effectiveness or superiority over structured health education (8–10).

Consequently, developing safe, scalable and evidence-based non-pharmacological strategies to enhance and sustain physical function and quality of life in individuals with knee osteoarthritis is now widely recognized as a high-priority research area, as emphasized in recent OARSI and EULAR guidelines for the non-surgical management of knee OA (11, 12).

While high-quality evidence underscores aerobic exercise as a cornerstone treatment for KOA due to its efficacy in improving cardiopulmonary function and weight management (13), this study prioritizes Tai Chi and Baduanjin for three critical reasons specific to the target demographic and holistic care goals. First, unlike pure aerobic regimens, these mind-body exercises integrate physical movement with mental regulation and breath control, offering unique dual-task benefits. Evidence suggests they provide additional advantages in improving balance (thereby reducing fall risk), enhancing psychological well-being, and developing pain coping mechanisms, which are vital for the comprehensive management of chronic KOA (14). Second, adherence is a pivotal challenge in exercise therapy for older adults. For the specific population affected by KOA—often characterized by fear of falling or limited mobility—mind-body exercises frequently demonstrate higher long-term adherence rates compared to strenuous aerobic protocols (15). Finally, given our research focus on Traditional Chinese Medicine (TCM) non-pharmacological therapies, investigating these culturally embedded practices is essential. They represent scalable, community-based interventions that are readily accepted by the aging population in Asia and are gaining global recognition.

Previous research has established that Tai Chi—an integrative mind-body modality that harmonizes deep diaphragmatic breathing, progressive relaxation, and deliberate yet graceful physical maneuvers—exerts beneficial effects on the health profiles of individuals with chronic diseases (14, 16, 17). Empirical evidence has documented substantial enhancements across a range of domains, including equilibrium, muscular strength, cardiopulmonary efficiency, physiological flexibility, pain mitigation, mood states, and attenuation of arthritis-related manifestations (18–20).

Recent studies have highlighted the therapeutic potential of Baduanjin in alleviating symptoms of osteoarthritis. By promoting fluid circulation within the joints and stimulating the surrounding musculature, Baduanjin can reduce stiffness and pain, thereby improving overall joint function (21, 22). Moreover, its low-impact nature minimizes the risk of further joint damage, making it a safe and accessible option for patients. Preliminary findings suggest that regular practice of Baduanjin can lead to significant improvements in physical function and quality of life for those affected by osteoarthritis, positioning it as a promising complementary therapy in the management of this chronic condition (23–25).

Hence, we are presently conducting a comparative efficacy and cost-effectiveness trial within a substantial cohort of patients with symptomatic knee OA.We propose that Baduanjin may offer distinct advantages over routine health education commonly delivered in clinical practice. Additionally, we seek to evaluate the comparative merits of Tai Chi in this context. Our overarching goal is to elucidate whether Baduanjin and Tai Chi can effectively reduce pain and functional limitations associated with knee OA, thereby enhancing the quality of life for individuals affected by this condition.

In this article, we outline the design and comprehensive protocol for the first three—arm randomized controlled trial comparing the clinical effectiveness and cost—effectiveness of Tai Chi, Baduanjin, and a health—education program in older adults with knee OA. We anticipate that this study will bridge significant knowledge gaps and provide valuable evidence to guide clinical practice and policy decisions related to the management of knee OA. The findings will be disseminated upon study completion, adhering to the Consolidated Standards of Reporting Trials (CONSORT) guidelines (26).

## Methods/design

### Study design overview

**Figure 1:**
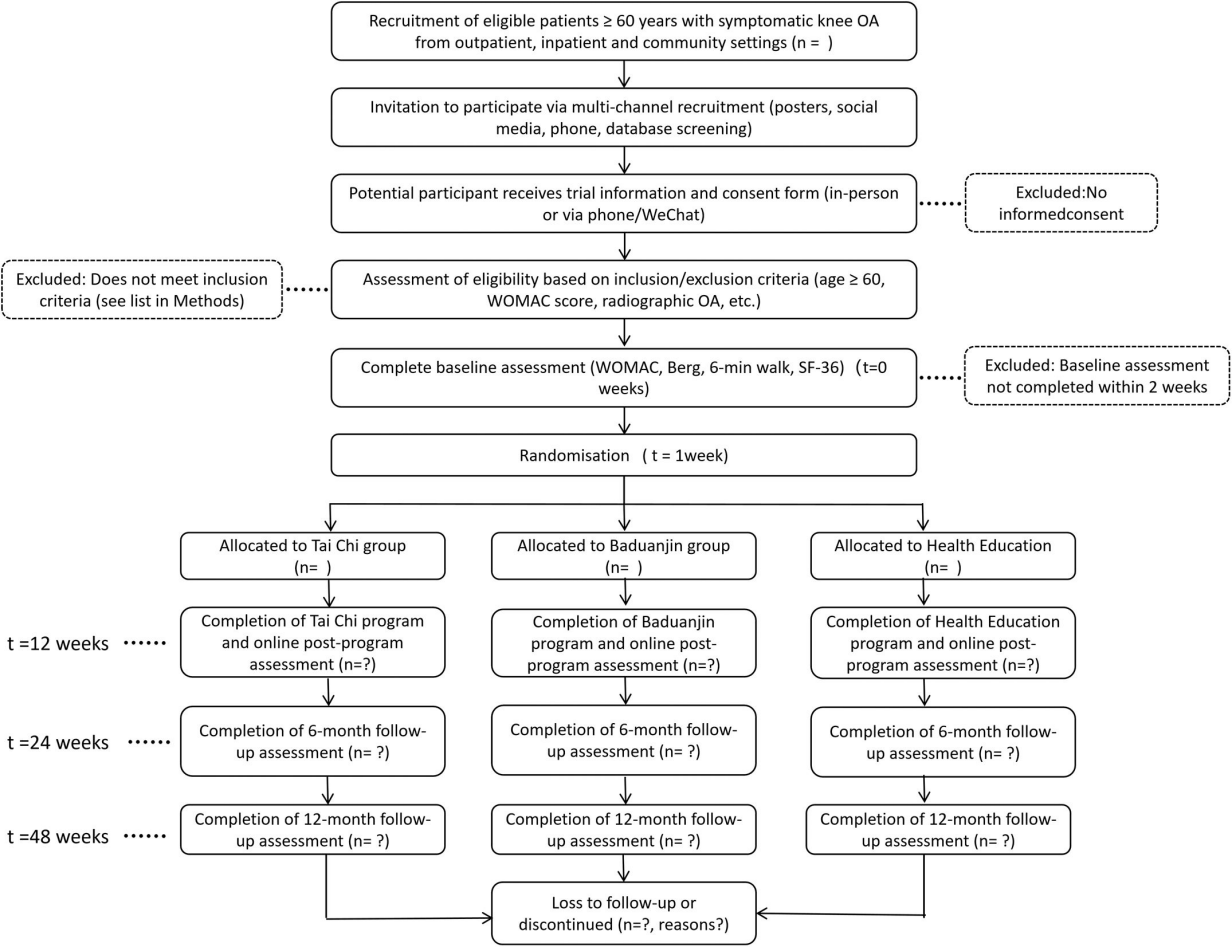
Study flow diagram.

This investigation is a single—center, 48-week randomized controlled trial (Figure 1). Study flow diagram). The design and reporting of this study protocol adhere to the Standard Protocol Items: Recommendations for Interventional Trials (SPIRIT) guidelines. A total of 120 older adults with symptomatic knee osteoarthritis will be randomly allocated to one of three groups: Tai Chi training (5 sessions per week for 12 weeks), Baduanjin training (5 sessions per week for 12 weeks), or a health-education program (1 session per week for 12 weeks), with 40 participants in each group.

The primary outcomes encompass the pain, stiffness, and physical function subscales of the Western Ontario and McMaster Universities Arthritis Index (WOMAC) (27). Secondary outcomes will be assessed using the Berg Balance Scale, which evaluates balance performance through a series of functional tasks; the 6-minute walk test, which measures endurance and mobility by assessing the distance walked in 6 min; and the Health Survey Short Form (SF-36), a comprehensive questionnaire that assesses overall health-related quality of life across multiple domains. These measures will provide a holistic view of the participants' physical and psychosocial well-being. Outcome measurements will be conducted at baseline, immediately following the 12-week intervention period, and at 24 and 48 weeks to assess the durability of treatment effects. The personnel administering the physical function evaluations and the data analyst are unaware of the participants' treatment allocations. The specific endpoints and their categorization as outcomes are detailed in Table 1.

**Table 1:** Schedule of assessments for primary and secondary outcomes.

Time	Baseline	Week 12	Week 24	Week 48
Primary Outcome variables				
WOMAC^a^—Pain	×	×	×	×
WOMAC—Physical Function	×	×	×	×
WOMAC—Stiffness	×	×	×	×
Secondary outcome variables				
SF-36^b^	×	×	×	×
Berg Balance Scale^c^	×	×	×	×
6 min walk test^d^	×	×	×	×

a WOMAC: Western Ontario and McMaster Universities Osteoarthritis Index.

b SF-36: Medical outcome survey short-form 36.

c Berg Balance Scale: A 14-item test that measures a person's balance and walking abilities.

d 6 min walk test: A simple test that measures how far a person can walk in six minutes.

The study setting is the Zhongshan Hospital of Traditional Chinese Medicine, affiliated with Guangzhou University of Traditional Chinese Medicine, located in Zhongshan, Guangdong, China. The study received ethics approval from the Institutional Review Board of Zhongshan Hospital of Traditional Chinese Medicine.

## Study status and timeline

As of the date of manuscript submission, participant recruitment has not yet begun. Recruitment is expected to commence in January 2026, with an estimated completion by June 2026. Data collection is anticipated to conclude by mid-2027, and the final results are expected to be available for analysis by late 2027. No data have been analyzed or reported at this stage.

### Study sample

The inclusion criteria for this study are as follows: Participants must be aged 60 years or older and have experienced pain symptoms for at least three months. They must be patients with mild to moderate osteoarthritis, as evidenced by a Western Ontario and McMaster Universities Osteoarthritis Index (WOMAC) pain subscale score ranging from 13 to 38 points (on a 0–50 points scale). Additionally, participants must have a total WOMAC score ranging from 50 to 150 points (on a 0–240 points scale) (28). Participants must be cognitively intact, with the ability to communicate effectively through writing or speech. They should also be able to walk independently without the use of assistive devices and be capable of performing a moderate amount of exercise on their own. Finally, participants must voluntarily agree to take part in this study and provide informed consent.

Participants will be excluded if they have any of the following: (1) prior experience with Tai Chi, Qigong, yoga, or similar exercises, or physical therapy for knee osteoarthritis within the past year; (2) severe medical conditions such as heart failure, malignant tumors, severe psychiatric disorders, or recent stroke; (3) history of trauma or ligament injury, or joint replacement surgery within the past six months; (4) severe joint deformity or inability to perform physical activity due to acute symptoms; (5) pharmacological or physical therapy for knee osteoarthritis within the past month; (6) regular exercise programs (≥30 min per session, ≥3 times per week); (7) inability to pass the Mini-Mental Status Examination (score < 24); (8) inability to walk without assistive devices; or inability to communicate in English; (9) pregnancy or plans for pregnancy; (10) participation in other clinical trials within the past 20 days; (11) or plans to relocate during the study period.

### Radiographs

Knee radiographs, including anterior-posterior (AP), lateral standing, and sunrise views, are captured during the initial screening phase (baseline assessment) in accordance with the Framingham study protocol (29). These images are assessed by the study's orthopedic expert to identify the presence of a definite osteophyte. If a prior clinical knee radiograph is available and a definite osteophyte can be clearly discerned by the study physician, substitution is allowed, even if the radiograph was obtained using a different protocol.

The weight-bearing knee radiographs are evaluated using the Kellgren-Lawrence (K/L) classification system to assess the overall severity of tibiofemoral joint degeneration. This system assigns a grade to each knee compartment, considering factors such as osteophyte presence, joint space narrowing, and subchondral sclerosis (30). The reported scores reflect the condition of the most severely affected knee, designated as the study knee. Additionally, the radiographs are assessed in accordance with the Osteoarthritis Research Society International (OARSI) atlas, which provides a detailed evaluation of osteophyte size and joint space narrowing in both the tibiofemoral and patellofemoral compartments (31).

### Knee examination

Evaluates the existence and degree of knee joint irregularities pertinent to knee osteoarthritis (OA) and the feasibility of safely engaging in the study. This includes assessing ligamentous instability, meniscal irregularities, flexion contractures, alignment deviations, and surgical scars.

### Recruitment strategies

To recruit participants, we implemented a multifaceted approach that included distributing informational flyers within hospital premises, posting recruitment notices on major social media platforms (e.g., WeChat and Weibo), and placing advertisements in local newspapers and community bulletin boards. We further expanded our reach by screening the patient databases of our hospital's orthopedic and rheumatology departments to identify eligible candidates. Additionally, we partnered with community organizations and established recruitment booths at health expos and senior activity centers to provide detailed information and address inquiries from potential participants.

### Enrollment and the informed consent process

Participants are enrolled in groups of 40 to ensure adequate sample sizes for each intervention arm. Baseline assessments are conducted for pre-screened participants three weeks prior to intervention commencement to secure an eligible cohort. Informed consent is obtained by the principal investigator or study coordinator before any data collection. Prospective participants are fully informed about the study, including objectives, procedures, risks, and benefits, and provide written consent.

Following informed consent, participants undergo a final eligibility verification. Those confirmed eligible are randomized into one of the three groups: Tai Chi, Baduanjin, or health education, using a computer-generated sequence. This ensures balanced group assignment and maintains the trial's integrity. A total of 120 participants will be randomized, with 40 in each group, providing sufficient statistical power to detect meaningful differences.

## Randomization and allocation concealment

To ensure the fairness and transparency of the allocation process, a computer-generated random sequence will be used to assign participants to the three intervention groups: Tai Chi, Baduanjin, and health education. The randomization sequence will be generated by an independent statistician who is not involved in participant recruitment or assessment.

Allocation concealment will be strictly maintained prior to enrollment. The randomization list will be kept in a secure, centralized system accessible only to the designated research coordinator responsible for enrollment. Until the moment of assignment, both the participants and the recruiting researchers will remain unaware of the upcoming group allocation to prevent selection bias. Participants will be randomly allocated to each intervention group with a target sample size of 40 participants per group.

Prior to randomization, all participants will undergo a detailed baseline assessment, including the pain, stiffness, and physical function subscales of the Western Ontario and McMaster Universities Osteoarthritis Index (WOMAC), as well as the Berg Balance Scale, 6-minute walk test, and SF-36 quality-of-life assessment. These baseline data will be used for subsequent statistical analyses to evaluate the effects of the interventions.

After allocation, participants will be officially enrolled into their respective study groups and begin the corresponding interventions. We acknowledge that due to the nature of these behavioral interventions, participants and instructors cannot be blinded to the group assignment after randomization. Therefore, rigorous measures have been implemented to blind outcome assessors and data analysts, as detailed in the following section. Throughout the study, we will closely monitor participants' adherence and intervention fidelity to ensure the accuracy of the results.

### Blinding

While allocation concealment is strictly maintained until the moment of randomization, we acknowledge that blinding of participants and intervention instructors is not feasible due to the distinct nature of the interventions (physical exercise vs. health education). To mitigate the potential performance and detection biases arising from this limitation, we have implemented rigorous blinding protocols for outcome assessment and data analysis. Specifically, all research staff responsible for follow-up assessments at weeks 12, 24, and 48 will be strictly blinded to the participants' group allocation; these assessors will not be involved in intervention delivery and will be instructed neither to inquire about nor allow participants to reveal their group identity.

Furthermore, the statisticians performing the final data analysis will remain blinded to the specific group codes until the statistical analysis plan is finalized and the primary analysis is completed. To further minimize performance bias from unblinded instructors, all intervention sessions will adhere to strictly standardized manuals with neutral scripts, ensuring that encouragement remains consistent across all groups without comparative claims. Finally, the success of these blinding procedures will be evaluated at the trial's conclusion by asking assessors and participants to guess the group allocation, allowing for the calculation of a blinding index to quantify the extent of any potential unblinding.

### Study intervention

The study interventions are designed to compare the clinical effectiveness of Tai Chi, Baduanjin, and a health-education program in older adults with knee osteoarthritis. The interventions will be conducted concurrently to avoid seasonal influences on disease severity. The primary goal is to assess the impact of these interventions on pain, stiffness, physical function, and overall quality of life over a 48-week period.

During the initial 12-week period, participants in the Tai Chi and Baduanjin groups will receive supervised group training sessions, while those in the health-education group will attend weekly sessions focused on educational content related to knee osteoarthritis management. After the 12-week intervention period, participants in all groups will be followed up at Weeks 24 and 48 to evaluate the long-term effects of the interventions. There will be no additional structured intervention programs during the follow-up period; however, participants will be encouraged to continue practicing the exercises they learned during the initial 12 weeks on their own.

### Tai Chi intervention

Participants assigned to the Tai Chi group will engage in a structured training program led by experienced instructors with over five years of teaching experience. The intervention will be based on a standardized “Simplified Tai Chi” protocol, specifically adapted for individuals with knee osteoarthritis. Over the course of 12 weeks, participants will attend five 60 min sessions per week, focusing on ten selected movements designed to improve balance, flexibility, and overall physical function without excessive joint stress. These movements include the Preparatory Stance, Rolling Back, Grasping the Knee and Twisting Step, Parting the Wild Horse's Mane, Cloud Hands, Golden Rooster Stands on One Leg, Toe Kick, Embracing the Sparrow's Tail, Cross Hands, and Closing Stance (32–34). Each session will begin with a 10 min warm-up, followed by practice of the Tai Chi movements, breathing techniques, and a 10 min cool-down period with relaxation methods. In addition to the supervised sessions, participants will also attend a weekly health education lecture for 12 weeks to further support their understanding of knee osteoarthritis management.

To ensure the quality and consistency of the intervention, all Tai Chi instructors will follow a standardized teaching protocol and receive training on the specific needs of individuals with knee osteoarthritis. Participants will be encouraged to practice Tai Chi at home for at least 20 min per day, with weekly assignments provided by the instructors to reinforce learning. Adherence to the home practice will be monitored through weekly feedback forms, and the quality of the sessions will be regularly assessed through video recordings and instructor feedback. After completing the 12-week intervention, participants will be asked to continue practicing Tai Chi independently, with monthly follow-up calls from the research team to monitor adherence and provide support as needed. Follow-up assessments will be conducted at Weeks 24 and 48 to evaluate the long-term effects of the Tai Chi intervention on pain, stiffness, physical function, and quality of life.

### Baduanjin intervention

Participants in the Baduanjin group will be led by experienced instructors through a 12-week program focusing on ten specific movements of the Baduanjin exercise routine. This traditional Chinese practice, known as “Health Qigong … Baduanjin”, is standardized by the General Administration of Sport of China. The series includes the following movements: Preparatory Posture, Holding Up the Three Burns with Both Hands, Left-Right Bow and Arrow Stance, Regulating the Stomach and Spleen by Raising Hands, Seven Injuries and Laboring to Look Back, Weaving Fingers to Strengthen the Waist, Clenching Fists and Angry Gaze to Enhance Energy, and Seven Injuries and Laboring to Look Back (35–37). Each session will start with a 10 min warm-up, followed by 40 min of Baduanjin practice, and conclude with a 10 min cool-down period to ensure proper form and technique are maintained throughout the exercise.

To maintain the quality and consistency of the Baduanjin intervention, instructors will follow a standardized protocol and provide weekly assignments to reinforce home practice. Participants will practice Baduanjin daily for at least 20 min, with adherence monitored through weekly feedback. Video recordings and instructor feedback will regularly assess session quality. Both the Tai Chi and Baduanjin groups will attend weekly health education lectures. After the 12-week intervention, participants will continue practicing independently, with monthly follow-ups from the research team. Follow-up assessments at Weeks 24 and 48 will evaluate the long-term effects on pain, stiffness, physical function, and quality of life.

### Health education intervention

The Health Education intervention, designated as the control group, will focus on providing participants with essential knowledge about knee osteoarthritis and general health management. Unlike the Tai Chi and Baduanjin groups, this group will not receive any standardized exercise regimen but will instead participate in weekly health education lectures. These sessions, each lasting 60 min with a 45 min presentation followed by a 15 min discussion, will cover a range of health-related topics. To ensure content standardization, the curriculum is divided into three core modules: (1) OA Management (pathophysiology and self-care); (2) Lifestyle & Exercise Science (principles of resistance, aerobic, and flexibility training, and obesity management); and (3) Comorbidity Prevention (management of diabetes, hypertension, and seasonal dietary practices).

The goal of this intervention is to ensure that all participants, regardless of their assigned group, receive a baseline level of health education, which can help them make informed decisions about their health and lifestyle. This approach allows for a balanced comparison of the additional benefits of the standardized exercise programs in the Tai Chi and Baduanjin groups.

### Measurements

The outcome measures for our study are derived from the recommended core set by the Osteoarthritis Research Society International (27), focusing on pain, physical function, and patients' overall assessment of their knee osteoarthritis severity. Participants will be assessed at baseline (prior to the intervention), immediately after the intervention (12 weeks later), and at 24 and 48 week follow-ups (Table 1).

## Statistical analysis

All statistical analyses will be performed using SPSS Statistics version 26.0 (IBM Corp). Continuous variables will be first assessed for normality using the Shapiro–Wilk test and for homogeneity of variance using Levene's test. Data presenting a normal distribution will be expressed as mean ± standard deviation (SD). For comparisons among the three groups, one-way Analysis of Variance (ANOVA) will be used. If the ANOVA indicates a significant difference, *post-hoc* pairwise comparisons will be conducted using the Bonferroni correction to adjust for multiple testing. Non-normally distributed variables will be presented as median (interquartile range, IQR) and analyzed using the Kruskal–Wallis H test, followed by Dunn's test with Bonferroni correction for pairwise comparisons if significant. Categorical variables will be expressed as frequencies (percentages) and compared using the Chi-square test or Fisher's exact test where expected cell counts are less than 5.

For the primary outcome (change in clinical scores from baseline to post-intervention), repeated measures ANOVA will be employed, with Group as the between-subject factor and Time as the within-subject factor. If a significant interaction effect (Group × Time) is observed, simple main effects will be analyzed to determine differences between specific groups at each time point, with Bonferroni adjustments applied for multiple comparisons. A two-sided *p*-value < 0.05 will be considered statistically significant.

### Primary outcome

The primary outcomes of our study are the changes in the Western Ontario and McMaster Universities Osteoarthritis Index (WOMAC) subscales for pain, stiffness, and physical function. These subscales are assessed at baseline and followed up at 12, 24, and 48 weeks post-intervention. The WOMAC is a widely recognized tool for evaluating knee and hip osteoarthritis, consisting of 24 items distributed across the three subscales (38). The pain subscale includes five questions scored from 0 to 10, summing up to a total of 0–50 points. The stiffness subscale comprises two questions, also scored from 0 to 10, with a total score range of 0–20. The physical function subscale contains seventeen questions, each scored from 0 to 10, resulting in a total score from 0 to 170. The total WOMAC score ranges from 0 to 240, with higher scores reflecting more severe disease. This comprehensive evaluation will allow us to assess the immediate and long-term effects of the interventions on the symptoms and functional limitations associated with knee osteoarthritis.

### Secondary outcomes

The secondary outcome measures in our study comprise the Health Survey Short Form (SF-36) (39), the Berg Balance Scale (40, 41), and the 6 min walk test (42, 43). These assessments aim to provide a detailed evaluation of various aspects of health and function related to knee osteoarthritis. The SF-36 is utilized to assess health-related quality of life across multiple dimensions, offering a broad perspective on the participants' general health status. The Berg Balance Scale evaluates balance and walking abilities, which are crucial for determining functional independence and fall risk in older adults. The 6-minute walk test measures endurance and mobility, providing insights into the participants' physical capacity and activity levels. Detailed descriptions of these measures and their specific application within our study are provided in the following sections.

The 36-Item Short Form Health Survey (SF-36) is a multidimensional health measurement instrument comprising nine scales and 36 items, designed to assess eight health domains: Physical Functioning (PF), Role Physical (RP), Bodily Pain (BP), General Health (GH), Vitality (VT), Social Functioning (SF), Role Emotional (RE), and Mental Health (MH). Additionally, there is a single item assessing Health Transitions (HT), which reflects perceived changes in health status compared to one year prior; this item is not included in the scoring of the subscales or the overall summary measures, but rather provides insight into the dynamic changes over time.

The SF-36 can be self-administered, administered by a trained interviewer, or completed via telephone. The typical completion time ranges from 5 to 10 min, although it may take up to 15 min for elderly participants. Scoring involves a weighted sum of item scores within each subscale to produce a raw score, which is then transformed into a standard score ranging from 0 to 100. Higher scores on the SF-36 indicate better health-related quality of life.

The Berg Balance Scale (BBS) is a widely utilized, reliable, and valid instrument designed to assess balance and functional mobility in individuals with various health conditions, including those with musculoskeletal disorders, neurological impairments, and the elderly. Comprising 14 items, the BBS evaluates a range of activities from sitting to standing, transferring, and walking, each scored on a 0–4 scale, where 0 indicates the inability to perform the task and 4 signifies the ability to perform the task independently without any assistance. The total score ranges from 0 to 56, with higher scores reflecting better balance and functional mobility.

The Berg Balance Scale (BBS) is an especially pertinent tool in the context of osteoarthritis, significantly aiding in the identification of patients at heightened risk for falls and monitoring advancements in balance-oriented rehabilitation programs. This scale is not only efficient to administer, typically taking 20–30 min, but also versatile, making it appropriate for diverse settings such as clinical assessments, rehabilitation facilities, and even home-based evaluations. Its responsiveness to change is particularly advantageous for appraising the impact of therapeutic interventions that target balance enhancement and fall risk mitigation in individuals with osteoarthritis. By providing a sensitive measure of balance and functional mobility, the BBS plays a crucial role in the comprehensive management of patients with this chronic condition.

The 6 min walk test (6MWT) is a straightforward yet robust assessment tool for evaluating an individual's functional exercise capacity and endurance. It entails having participants walk as far as possible along a flat, straight course within a six-minute timeframe. This test is especially valuable for assessing patients with conditions that impair mobility, such as osteoarthritis, heart failure, and pulmonary diseases. Conducted in a controlled environment with minimal distractions, the 6MWT is a safe, low-cost procedure that seamlessly integrates into clinical settings, rehabilitation programs, and research. It measures the distance walked, which corresponds to the patient's exercise capacity and provides insights into their ability to perform daily activities. The test is well-tolerated and offers a practical means of gauging a patient's physical capabilities.

Moreover, the 6MWT is sensitive to changes over time, rendering it an excellent tool for monitoring the effectiveness of interventions like physical therapy, medication, and lifestyle changes. Its reliability and validity have been established across diverse patient populations, including those with osteoarthritis. The 6MWT helps in assessing how disease and treatment influence physical function and overall quality of life, making it an indispensable part of comprehensive patient care and outcome evaluation.

### Adherence

Ensuring participant adherence to the study interventions is critical for the validity of our findings. To enhance compliance, we will implement a multifaceted strategy. For the Tai Chi and Baduanjin groups, attendance will be meticulously tracked during the initial 12-week supervised sessions, with personalized feedback from instructors to boost engagement. Home practice will be encouraged, monitored via weekly feedback forms, and reinforced with monthly follow-up calls post the intervention period. The health-education group will engage in weekly interactive sessions to sustain interest. All participants will receive regular adherence reminders and incentives at key study milestones to acknowledge their contributions.

Post-intervention, adherence will be assessed through monthly phone calls using standardized questionnaires, focusing on the frequency and duration of exercises. This approach will provide a comprehensive view of long-term engagement with the interventions, crucial for evaluating the sustainability of the study effects.

### Safety

Throughout the study, participant safety is of paramount importance. We will conduct weekly assessments during the intervention phase to identify any adverse events, which are defined as any unfavorable or unintended signs, symptoms, or medical conditions. Each adverse event will be meticulously documented on a standardized case report form and subsequently evaluated by the study's rheumatologist. This evaluation will determine the relevance of the event to the intervention and its severity, adhering to the criteria mandated by the Institutional Review Board (IRB).

The recorded adverse events will be categorized and analyzed for any patterns that may suggest potential safety risks to participants. Special attention will be given to serious adverse events and those considered related to the study interventions. Safety monitoring will be conducted throughout the entire study duration, from baseline through the final follow-up at week 48. While intensive monitoring occurs during the 12-week intervention phase, systematic surveillance for adverse events (AEs) will continue during the post-intervention follow-up periods (weeks 24 and 48).

This safety monitoring plan has been reviewed and approved by the ethics review board and an independent data and safety monitoring board, ensuring that the study adheres to the highest standards of participant safety and ethical conduct.

### Data management

In our study, data will be systematically collected and managed through the hospital's internal clinical information system (CIS), which is designed to handle sensitive medical data with high security standards. For participants who are unable to directly input their data into the CIS, paper-based case report forms will be utilized. These forms will be completed by the participants and subsequently entered into the CIS by trained study staff members. The entered data will be verified for accuracy and completeness before being stored securely in the participants' electronic files, which will be kept in a restricted access area within the hospital's information system.

To ensure data integrity and confidentiality, no personal identifiers will be included in the study database. Data will be extracted from the CIS and transferred into statistical software for analysis, ensuring that the process adheres to strict privacy protocols. The CIS also features an audit trail function that logs every instance of data modification, whether by participants or staff, providing a transparent record of all changes made to the data entered into the system.

This approach to data management not only ensures the security and confidentiality of participant information but also facilitates efficient data processing and analysis, which are essential for the reliability and validity of our study outcomes.

### Sample size

We aim to recruit a total of 120 participants, evenly distributed across three intervention groups, with 40 participants in each group. The determination of this sample size is based on projected improvements in key outcome measures, including the Western Ontario and McMaster Universities Osteoarthritis Index (WOMAC) for pain and physical function, as well as the physical component summary of the SF-36 health survey. Our calculations are informed by the findings from relevant randomized controlled trials (RCTs) that have utilized active control conditions, which provide a robust basis for estimating the anticipated treatment effects. This sample size is anticipated to provide sufficient statistical power to detect meaningful differences in outcomes between the intervention groups while accounting for the within-study variability.

The sample size was calculated using G*Power software (version 3.1) for a one-way analysis of covariance (ANCOVA) to handle the three-group comparison, adjusting for baseline values of the primary outcome (change in WOMAC score at week 12). Key statistical parameters were determined based on findings from relevant randomized controlled trials utilizing active controls, specifically studies by Hu et al. (44) and Ye et al. (45). Effect Size (f): Given the large and robust improvements in proprioception and WOMAC function scores (*p* < 0.05) consistently reported in these studies, we anticipated a large between-group effect size of *f* = 0.32. This corresponds to an expected mean difference of approximately 5.0 points with a common standard deviation (SD) of 9.0 points. Significance Level (α): Set at 0.05 (two-tailed). Statistical Power: Set at 80% (0.80).We are conservatively factoring in a 15% dropout rate to ensure the study retains sufficient statistical power. This adjustment helps to maintain the robustness of our findings even if a portion of the participants withdraw from the study.

In the event that the dropout rate exceeds our projected figures, we plan to perform a reassessment of the statistical power to ascertain the necessity of enlisting additional participants. This reassessment is crucial to ensure that the study maintains adequate power to detect the intended treatment effects. Moreover, if it is determined that an increased sample size is essential for enabling robust comparisons among participant subgroups and for ensuring equitable group dynamics, we will undertake a reevaluation of the sample size. This process is integral to preserving the methodological integrity of the study and to substantiating the generalizability of our findings.

### Analysis

The primary outcomes for this study are defined as the changes from baseline to Week 12 in the three WOMAC subscales: Pain, Stiffness, and Physical Function. The primary analysis will be conducted using an intention-to-treat approach, where all participants are analyzed in the group to which they were randomized, without adjustment for covariates, and with missing 12-week outcomes imputed as no change. Secondary analyses will adjust for baseline characteristics that differ significantly by treatment group, including gender, initial pain severity, medication use, disease duration, and the number of chronic conditions.

Changes in knee pain at weeks 24 and 48, along with all other outcomes, are considered secondary and will be analyzed in the same manner as the primary outcome. We will also perform a longitudinal analysis across all four time points to identify potential time trends and interactions between treatment effects and time. These analyses will utilize mixed-effects models with random intercepts for participants and fixed effects for time, considering autoregressive structures for residual errors.

To handle missing values, we will employ multiple imputation techniques under the assumption of missing at random (MAR). Given the potential for early dropout due to poor outcomes, we will also explore models that account for missing not at random (MNAR) based on informative dropout assumptions. As part of our sensitivity analyses for missing data, we will consider Bayesian models that can incorporate prior assumptions about the non-ignorable missing data mechanisms.

All statistical tests will be two-tailed, with a significance level set at *p* < 0.05. We will conduct both individual time-point analyses and longitudinal analyses to provide a comprehensive understanding of the treatment effects over time.

### Cost-effectiveness analysis

Our study will conduct an economic evaluation to assess the cost-effectiveness of Tai Chi, Baduanjin, and health education for knee osteoarthritis management. We will focus on direct medical costs like intervention expenses, medications, and hospital stays, as well as indirect costs such as productivity losses. Quality-adjusted life years (QALYs) will be calculated using SF-36 utility scores to measure health state utilities. The cost per QALY gained will be the primary economic outcome, analyzed over the trial duration and in a projected extension.

To estimate long-term health and economic implications, we will use computer simulation models, including decision analysis to evaluate outcomes under uncertainty. This process will involve a literature review for model parameters and the development of Markov and Monte Carlo simulation models based on trial data. The cost-effectiveness analysis will present incremental cost-utility ratios, allowing comparison with accepted healthcare decision-making thresholds. Sensitivity analyses will be conducted to assess the impact of key parameter variations on the interventions' cost-effectiveness, ensuring a robust economic evaluation.

## Discussion

Our study introduces a randomized controlled trial featuring a distinctive three-arm design to assess the clinical efficacy of Tai Chi, Baduanjin, and health education for individuals with knee osteoarthritis. This approach allows for a direct comparison among these interventions, providing a clearer understanding of their relative benefits and potential as alternatives or complements to conventional treatments.

The inclusion of both traditional exercises and health education within the trial framework ensures a thorough evaluation of non-pharmacological strategies for knee osteoarthritis. By directly comparing Tai Chi and Baduanjin, our study aims to ascertain whether one intervention is superior or if they offer similar benefits, which is essential information for both patients and healthcare providers.

The results of this study are anticipated to enrich the evidence base for managing knee osteoarthritis through non-pharmacological means. The comprehensive clinical evaluation of these interventions is expected to yield valuable insights into their practical utility and effectiveness in improving patients' quality of life. This study's findings could support more informed decision-making in clinical practice, potentially influencing treatment guidelines and patient management strategies for knee osteoarthritis.

A primary limitation of this study is the imbalance in intervention frequency and contact time between the exercise groups (5 sessions/week) and the health education control group (1 session/week). This design reflects clinical guidelines recommending frequent practice for mind-body exercises, whereas the control represents standard care. Consequently, observed differences may be attributed to the comprehensive intervention package (including increased clinician attention and time commitment) rather than the specific physiological effects of Tai Chi or Baduanjin alone. Future studies employing dose-matched active controls are needed to isolate the specific modality effects.

Furthermore, as is inherent to behavioral interventions, participants and instructors could not be blinded, introducing potential performance bias. To mitigate this threat to internal validity, we strictly maintained blinded outcome assessment throughout the 48-week follow-up and incorporated objective physical performance measures to complement self-reported data.

While intervention fidelity is rigorously maintained during the initial 12-week supervised phase through standardized protocols and trained instructors, the subsequent unsupervised follow-up period relies partially on participant self-report (e.g., exercise logs). This shift may introduce variability in intervention exposure. We have implemented periodic telephone checks and log reviews to monitor adherence, but the potential for fidelity decay in the long term remains a limitation common to pragmatic trials.

Finally, the external validity of our findings may be constrained by the single-center setting within a specific Chinese hospital and the high frequency of required exercise sessions. These factors may limit the generalizability of results to healthcare systems with different resources or to community-dwelling older adults who may struggle with high-frequency adherence. Our findings should thus be interpreted as the efficacy of these interventions under optimal, supervised conditions. Future research should focus on adapting these protocols for diverse cultural contexts and evaluating their effectiveness in real-world, lower-resource community settings.
